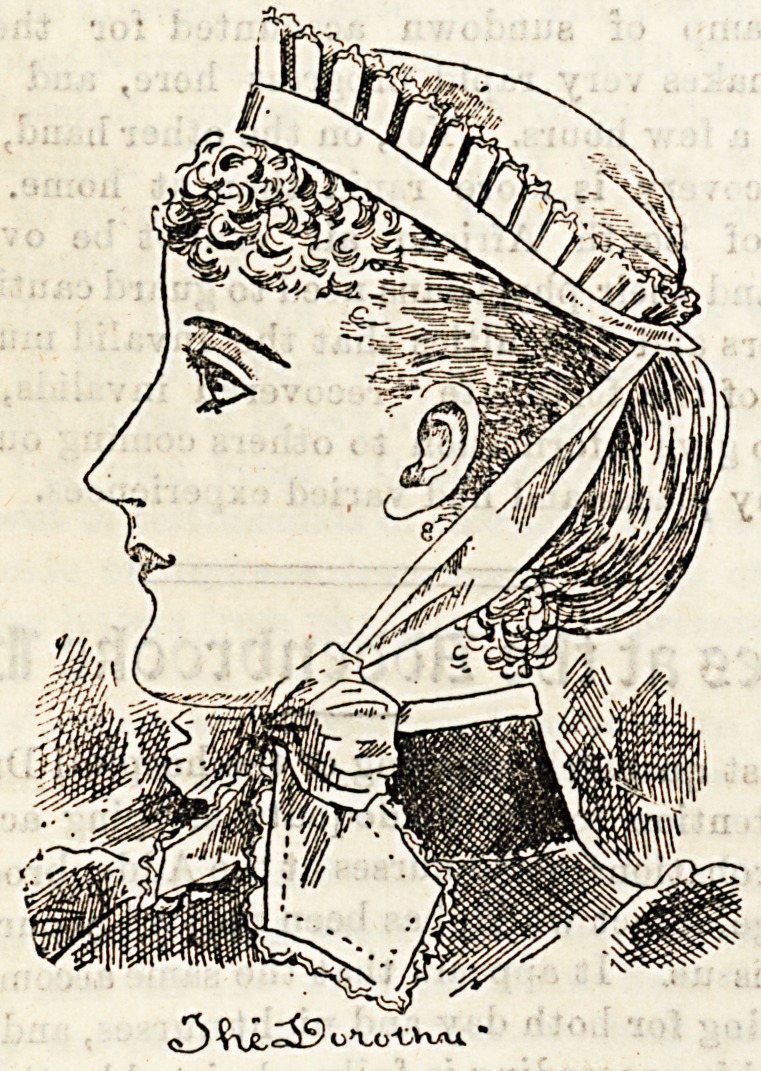# The Hospital Nursing Supplement

**Published:** 1894-10-20

**Authors:** 


### "The Hospital" Nursing Mirror.

Being the Extra Nursing Supplement of "The Hospital" Newspaper.

[Contributions for this Supplement should be addressed to the Editor, The Hospital, 428, Strand, London, W.C., and should have the word "Nursing" plainly written in left-hand top corner of the envelope.]

### News from the Nursing World.

### NURSING AT GUY'S HOSPITAL.

Much has been accomplished during the past twelve months towards improving the position of the nurses at Guy's Hospital, and their dietary has received due attention, the meals provided being satisfactory in quality and service. In and out door uniform are provided, as well as laundry, and the nurse's sick-room is a well-arranged department. It has been recently done up and partly re-furnished, with due regard to the tastes of the occupants. The substantial assistance given to probationers in taking out policies in the Royal National Pension Fund is not one of the least of the advantages which the authorities of Guy's offer to their nurses. It is also a tangible proof of the interest taken in the present and future good of the workers. Evidence of the good feeling existing between the medical and nursing department is shown by a notice in the October number of *Guy's Hospital Gazette* to the effect that nursing news is inserted with the consent of the Treasurer and Matron, and that the editors have promised not to put in communications until they have been officially approved by both

### THE ROYAL FREE HOSPITAL.

Better accommodation for the women students and better sitting rooms for the nurses are some immediate results of the rebuilding of the front of the Royal Free Hospital in Gray's Inn Road. There is said to be no present possibility of improving the construction of the nurses' dormitories, and it is therefore fortunate that the occupants are inclined to make the best of the existing arrangements. A four years' course of training is adopted at this hospital, and the probationers receive no payment during their first year, but get board, lodging, and washing; in the second year £15, in the third £20, and in the fourth £25 is the rate of salary given. Probationers may be willing to bind themselves for this period to a hospital which offers facilities for general training, but at the end of the time they will still require to devote six months to learning midwifery and fever nursing respectively if they aspire to be considered fully-trained nurses. The regulations of holidays and time off duty appear well arranged, and it is evident that the committee and matron have devoted much care to the elaboration of the rules which came into force last spring. Uniform is given after the three months' trial has expired; pupils have, however, to provide twelve aprons, three dresses, and three caps for themselves when they first enter the hospital.

### OVER THE HOSPITAL WALL.

The difficulties in obtaining capable nurses for fever hospitals do not appear to diminish. The matron of one of these institutions engaged as charge nurse a woman "calling herself Catherine Bassett." At the end of two months she resigned on the plea of being about to be married, but instead of finishing her time of service at the hospital, her mode of departure is thus described by the matron: "At dark, secretly, without warning or reason, she left her wards, full of scarlet fever patients, had her box taken from her room around the back of the wards, over the hospital wall to the station, and took the train for London." We should be glad to publish any explanation Catherine Bassett may have to offer of the causes which led her to take this journey of hers "over the hospital wall."

### DISTRICT NURSING AT STOCKPORT.

The Stockport Sick Poor Nursing Association had not a very good financial position to report at its third annual meeting. Whilst the record of work done appears excellent, the subscriptions do not reach the total necessary for the support of such a charity, which is purely unsectarian as well as practical. Even that powerful auxiliary, the Nourishment Fund, has an expenditure in excess of its receipts, an unfortunate circumstance when the extra demands made by winter on its funds are imminent.

### A USEFUL INSTITUTION.

The annual meeting of the Governesses' Convalescent Home for the North of England was recently held at Southport. About one hundred and thirty ladies passed through the home during the year, each making an average visit of a month. Structural repairs have formed rather a heavy but unavoidable item, but on the other hand an unexpected legacy of £1,000 was gratefully acknowledged by the committee.

### SICK PAUPERS IN THE NEW FOREST UNION.

According to the recent report of the Board there is not a single able-bodied female pauper in the workhouse in Lyndhurst Road, a condition of things which should be satisfactory to the New Forest Union Guardians, except for its bearing on the care of their sick and infirm inmates. Apparently these are without any night attendance, for the medical officer and the master agree as to the urgent need for someone to be put in charge at night. A wardswoman seems the only kind of helper suggested; an outside pauper, whose "relief" will be stopped whilst her temporary engagement lasts. According to the local press, skilled nursing for the sick paupers was not even suggested.

### IS NURSING PROFITABLE?

The question comes under all sorts of disguises, and often gets answered in the negative. "Only twenty pounds a-year!" exclaims a friend, as if the sum were beneath contempt, overlooking that a nurse in a hospital gets also board, lodging, washing, uniform, medical attendance, and, if she needs it, free nursing, and when fully trained and experienced higher pay and longer holidays fall to her lot. Truly she deserves all she earns by her steady work, but in many other callings a woman may work year after year for higher pay, and yet her "spending" money is actually on a far lower level, because nothing whatever is "found" for her. When she stops work through illness the attendant expense has to be met by her own slender resources, and her recovery is retarded by a terrible dread that her place may be filled before she is well enough to claim it. In few departments of women's work is there any possibility of a pension such as is given by the R.N.P.F. for Nurses, and that not as a chance or a charity, but as a certainty secured by foresight and thrift.

### ABERDEEN.

The annual meeting of the Banchory District Nursing Association took place last month, and the work of the Queen's Nurse was shown to be acceptable as well as valuable. The Inspector of the Scotch branch of the Queen Victoria Jubilee Institute also reported favourably of Nurse Myers' work. Mrs. Lumsden, by an opportune gift of furniture, facilitated the settlement of the nurse in comfortable rooms. Others have shown kindly interest, and many little offerings have been forthcoming to complete the furnishing and also to supply comforts for the sick. The Aberdeenshire branch of the Scotch Needlework Guild has given a grant and a supply of garments for lending to the patients. A letter was laid before the meeting expressing the sympathy of H.R.H. Princess Louise with the Banchory Association, and testimony was volunteered by several medical men as to the aid rendered in their practice by the trained nurse.

### NURSES IN FRANCE.

In comparisons recently made between lay nurses and nuns, those who advocate the employment of the latter in Paris hospitals apparently ignore the need for their being fully-trained nurses. If the women now engaged are as unsuited to responsible posts as is suggested, it is surely due to the patients that whether they be "lay" women or professed "sisters," they should be replaced by well-trained nurses.

### NURSES IN CEYLON.

The Ceylon Nurses' Association is making progress, several new features being introduced at the meeting in September at Darrawella. The committee seem sanguine that the Jubilee Fund will be eventually granted to them by the Planters' Association, as the Lady Havelock Hospital, to which it was promised, needs no further assistance. Amongst the rules of the Nurses' Association No. VI. arrests special attention from its originality. It runs, "that in the event of a married subscriber not being able to attend a meeting, his wife shall have power to attend and vote in his place, or, if the lady is a subscriber and cannot attend, her husband shall have power to vote in her place." Lady Havelock has been elected president, and Mrs. Edwards hon. sec. and treasurer in place of Mrs. Pole Carew, who is coming to England, and whose services in forming the association were duly acknowledged at the meeting.

### DISAPPOINTED BURGLARS.

At Agra, a few weeks ago, a daring burglary was committed. Some natives broke into the Chemical Laboratory there, and made a raid on some very carefully sealed glass bottles. Doubtless, they were much elated at getting safely away with their prize, for they believed that the fluid contained in these transparent receptacles was, indeed, beyond price. They could hardly believe in their good fortune when they found themselves out of sight and sound of the lawful owners of the glass bottles which they were convinced held the true Elixir of Life. Those who had been robbed however, lamented loudly that they had lost a collection of specimens of the true microbe of cholera!

### TRAINING FOR MALE NURSES IN U.S.A.

Although for seven years the school for male nurses at the New York City Hospital has existed, it has only presented its first report since Miss Louise Darche, Superintendent of the New York City Training School, commenced her responsibilities on March 1st, when she took over the nursing of the male wards, which contain a daily average of 350 patients. The Head Orderly was replaced by Miss Minnie S. Decker, a graduate of the N.Y.C.T.S., who was appointed Supervising Nurse. Washing uniform coats have been adopted, as well as regular weekly lessons, and a course of lectures by visiting physicians. Already a considerable improvement has been effected in the nursing of the patients and in the condition of the wards, and Miss Decker has been able to introduce many improvements. It is not difficult to foresee the extreme value of a progressive step which might, indeed, be advantageously followed up in other countries, where the male nurse asks vainly for systematic instruction.

### NURSES AT BOSTON.

The nurses' club at Boston, known as the Association of Trained Nurses, has been socially successful during its six months' existence. A scientific or nursing lecture is given occasionally, and all the medical and nursing journals are provided for the members. There are seven nurse training schools at Boston, and there are, it is stated, from six to seven hundred nurses connected with the "Registration Bureau," who have all had a certain amount of training. However, numbers of these have been for many weeks unemployed, and it seems as if the fees would have to be reduced to correspond with the diminished incomes of the citizens.

### SHORT ITEMS.

A ball was recently given at Penarth in aid of the Victoria Nurses' Institute.—At St. Mary's, Isles of Scilly, the annual meeting of the Dorrien-Smith Memorial Nursing Association took place the other day, and a good report of work was laid before the subscribers; Miss Alma Smith-Dorrien has been appointed President of the association.—The Westbury parish nurse continues to give satisfaction, and at the annual meeting was reported to have paid 1,424 visits to 85 patients in the course of last year.—The York Board of Guardians are taking steps to provide night attendants for the sick paupers.—On the 13th inst. a meeting of the Executive Committee of the Matrons' Council took place at St. Bartholomew's Hospital, in the Matron's house.—A meeting of the Matrons' Council is announced for Nov. 1st.—A Debating Society for the nursing staff at St. Bartholomew's Hospital has been organised by Miss Isla Stewart.—The continued success of the Nurses' Co-operation, 8, New Cavendish Street, was strikingly shown at the last general meeting, and this success has from time to time encouraged the formation of various societies on somewhat similar lines. Just now the demand for private nurses is not great, and at the committee meeting on the 9th inst. the Registered Nurses' Society decided that encouragement should not be given to workers to leave other branches for private nursing.

### Notes for Nurses on Antiseptic Surgery.

By William Horrocks, M.D., F.R.C.S., Hon. Surgeon to Bradford Infirmary.

### II.—TREATMENT OF THE PSOAS ABSCESS.

Perhaps there are no cases more troublesome to treat than the large abscesses which are connected with diseased bone in the spine (psoas abscesses). These abscesses, so long as they are unopened, remain comparatively harmless, but when once they are opened fever sets in, with sweats and wasting, causing death within a very short time.

### Abernethy's Method.

Early in this century Mr. Abernethy had gained a considerable reputation for curing these abscesses by sliding the skin, puncturing the abscess, allowing the pus to escape, then closing the cavity by sliding the skin back. In this way the air was prevented from gaining access to the abscess cavity.

### Stromeyer and the Tenotome.

Considerably later a German surgeon, Stromeyer, devised a method for dividing tendons by passing a narrow-bladed knife, a tenotome, under the skin, so by making the wound in the skin small and the wound of the tendon at a considerable distance from it, the air was excluded. Such wounds usually healed by first intention, without suppuration. Hence exclusion of air in surgery favoured healing by first intention, as shown by the gliding and subcutaneous method.

### Presence of Sepsis.

Briefly to recapitulate, Lister assumed that suppuration of wounds was due to their infection by a poison (sepsis), which was very widely spread and especially carried about by the air; that this material settled on the wound while the operation was performed; and that it was present on the hands of the operator, on the instruments, on sponges, and on the usual applications to wounds. He had thus proved the presence of the first of our three requisites, the sepsis or poison, and how it gained access to the wound.

### Infection of Blood Clot.

His next experiments were to show that blood clot readily became putrid if infected in this way, showing that this was the necessary medium for the ferment to grow on. (3) That the temperature of the body and the supply of moisture were favourable to the growth. His observations showed that living tissues resisted the growth of the septic material, but when extensively damaged formed a more suitable soil—*e.g.*, the small wound caused by the sharp edge of a razor seldom suppurates; the thin layer of destroyed tissue allows no resting-place for the septic material, and the sound tissue below resists their growth. On the other hand, extensive bruising or death of the part by killing a thick layer of tissue offers a suitable place for the septic material to grow.

### Discovery of Cause.

Lister had by his experiments and experience found the cause of wound suppuration; it remained to devise methods to prevent the infection of wounds.

### Prevention Suggested.

The first thing necessary was the discovery of agents which would kill the organisms of putrefaction, the next to employ means to prevent their access to wounds, and lastly to diminish the materials in which the organisms can grow.

### Carbolic Acid and Its Uses.

The substance first used to destroy the organisms of putrefaction was carbolic acid, which was greatly used by Lister. Carbolic acid is obtained by distillation from coal tar. As used in surgery, in the form of absolute phenol, it consists of white crystals. These crystals are caustic, *i.e.*, destroy the tissues, forming an eschar or slough. Carbolic acid is soluble in water, the usual strength of solutions being 1 part of carbolic acid dissolved in 20 parts of water, or 1 part of carbolic acid dissolved in 40 parts of water. These solutions should be kept in stoppered bottles, as carbolic acid is volatile, *i.e.*, it escapes as a vapour, if left exposed to the air. The solutions should be clear, presence of oily globules denotes impurity of the acid. Carbolic acid has a sweet taste, and produces a numbness of the skin, if immersed in it. Lister used the stronger solution 1 in 20 for washing the skin of the patient and the instruments, 1 in 40 was used for the hands of the operator. Carbolic oil, 1 part of carbolic acid dissolved in 10 parts of oil, was used for catheters.

### Lister's Early Procedure.

In the first days of this treatment, Lister's method of procedure may be described as follows:—

The skin around the part for operation having been thoroughly cleansed and any hair removed by shaving, was soaked in 1 in 20 carbolic acid solution. The surgeon's hands and instruments placed in carbolic acid lotion.

### Protection of Wound.

The wound was protected as much as possible even during the operation by a guard of lint soaked in carbolic acid, while all the time constant irrigation of the wound with 1 in 40 carbolic acid was kept up; the vessels were tied, and all bleeding stopped by catgut ligatures, with which the lips of the wound were brought into apposition. A rubber drain was inserted.

### The Dressing.

Lastly, the wound was covered with several folds of gauze steeped in 1 in 40 carbolic acid lotion, and to prevent evaporation a piece of lead foil was placed over this with its edges made to fit closely to the skin by putty made with carbolic oil. The lead plate was fixed in place by a bandage. This was Sir Joseph Lister's first method. It carried out his theory.

### Freed from Organisms.

The skin of the patient he freed from organisms by washing with carbolic lotion; the organisms were prevented from getting into the wound by screening it with a guard and constantly washing the wound with lotion. The vessels were carefully tied to diminish the amount of material for the organisms to grow in. The drainage tube allowed fluid to escape, which might undergo decomposition. The wound was kept surrounded by an atmosphere of carbolic acid, which was prevented from volatilizing by its covering of lead foil.

---

### Nurses as Sanitarians.

"Your tap is dripping," said Nurse Mary, as she entered the house of a patient in her district. "Yes, Nurse," said Mrs. Brown with an air of satisfaction, "it isn't only doctors and nurses who can tell us how to keep healthy. I never turns that tap off day nor night, and it's constantly a-drippin' and carrying off everything nasty with it. Jane sometimes complains of a smell in here, she do, but I tells her it can't be no harm as long as I keeps the tap a-drippin'." Nurse Mary had passed no degree in sanitary science, but she had acquired enough knowledge of the common sense of sanitation to check many an abuse and rectify many a mistake in the homes of the sick poor whom she visited. In this instance she found Mrs. Brown's mistaken waste of water had prevented a proper flushing of the waste pipe, and was the origin of the severe case of sore throat in the house at which she was attending. Mrs. Brown willingly learnt wisdom, as most Mrs. Browns will be found ready to do, when the teacher is kind and able.

### Nursing in Rio de Janiero.

BERI BERI.

I have recently visited the "Beri-Beri Hospital" at Copacabana Bay, and like all the hospitals in Rio de Janiero it is very pleasantly situated. Facing the South Atlantic, on the edge of a hill, with all that nature can supply in the way of beauty and pure air, it should be a perfect place, yet the nature of the disease destroys peace. I asked the doctor in charge for the actual meaning of "Beri-Beri" as so many versions are given, but even he could not tell me; he said it is a distinctly infectious disease without doubt, and that many movements are on foot to discover the real microbe, but up to the present without success.

There were fifty cases, in various stages, under treatment at the time of my visit, and from what I saw it appeared to be a bad mixture of Bright's disease and anæmia. One man of twenty-five, was quite puffy about the head and body, but both upper and lower extremities were shrunken and flabby; another man of forty was simply a swollen mass of humanity, quite unrecognizable by his nearest relation, and this is the first stage. After the swelling subsides, the muscles, more particularly those of the extremeties, become hypertrophied, and paralysis follows.

It occurred to me that this was a disease where massage would be most beneficial, so I asked if they tried it or the battery, but was told no; the chief treatment was good nutritious food, consisting chiefly of fish, eggs and chicken, douche baths, and plenty of exercise. The latter seemed rather a cruel sarcasm, for it was most painful to see the poor things struggling about on crutches with their shrunken limbs dangling helplessly; some were completely paralysed. If fresh air and plenty of exercise are the great things I could not help wondering how patients were to get better, but of course I did not wish to make myself objectionable by too many questions, having been so kindly and courteously treated at the hospital. Of course a nurse is a strange creature to some of the people here, and as the "Beri-Beri" question is already in such very able hands, it would be presumption to offer suggestions, but I most sincerely trust the time is not far off when means to secure more rapid recoveries may be looked for. At present it takes as long as twelve months to effect a cure—"from one to twelve" the doctor told me. The principal medicines used are zinc, arsenic, and quinine. The disease attacks white and coloured people from fourteen to fifty years of age. I also asked if it was due to living in marshy districts, but the doctor said that the patients came from different places; some of the officers and men who had taken part in the late revolution were under treatment, also men from the prisons.

Then I inquired if they got albuminuria, and was answered in the negative. Altogether it appears to be a most unsatisfactory disease, and one that is much dreaded, entire change of climate is the only remedy.

Although so prettily situated, this hospital is not so well appointed as others visited, and the reason may be because it is in permanent use, instead of only during an epidemic like the other infectious hospitals, thus involving more expense and the maintenance of a permanent staff, &c.

Cornstalk.

### Our American Letter.

The new Superintendent of the Training School for Nurses at Wilksbarre City Hospital, Wilksbarre, Pa., is Miss Mary W. McKeeling, of the Louisville Training School. She has received many congratulations on her appointment. The Flower Mission has given up its Training School at Indianapolis, having much other work on hand. The school, however, will not be allowed to lapse, but will continue as the Indiana School of Nursing, in connection with the Indianapolis City Hospital, under the superintendence of Miss Florence Hutchison. Thirteen coloured young women are being trained as nurses in the Dixie Hospital, where black as well as white patients are treated.

A Nurses' Home has been taken at Baltimore to receive some twenty of the members of the Nurses' Association in that city. Each nurse is to pay five dollars per month, and will furnish her own room. Qualifications for admission appear to consist in testimonials of character from a minister, and a certificate or diploma from some college or hospital school, and also letters from physicians as to private or institutional work.

A two years' course of instruction is offered to nurses at the new training school connected with the Cortland Hospital.

The Boston Training School has been at work for twenty years, and may well be congratulated on the success which it has achieved, and on the high standard held before the nurses. They are exhorted to "honour their profession," and to avoid the present tendency to regard skilled nursing primarily from a professional aspect, overlooking the requisite personal qualities. The Superintendent of the Boston School warmly advocates the best interests of the graduates, and gives great attention to their comforts. She has recently effected some important improvements in the commissariat, and the nurses have a late dinner

The American monthly journal, the *Trained Nurse*, contains some good articles in the October part, notably "Nurses' Work in Hospitals," by Miss Josephine Osborn, Superintendent of Aultman Hospital, Canton, Ohio. With regard to a private nurse, she says to employers: "Remember she is human, and has not been trained to do without food or sleep." And to the nurse herself she writes: "Carry conscience into the smallest details of your work. Be worthy of your certificate or burn it. . . . Nursing has in it a personal element that nothing can supersede. It will be just what the nurse herself makes it."

### Appointments.

[It is requested that successful candidates will send a copy of their applications and testimonials, with date of election, to The Editor, The Lodge, Porchester quare, W ]

General Hospital, Birmingham.—Miss Zara Stevenson has been elected Matron of this hospital. She was trained for three years at the Glasgow Royal Infirmary, and for three years held the post of Sister at the Royal Infirmary, Bristol. For the last three years Miss Stevenson has been Matron of the Rotherham Hospital, and she takes many good wishes with her to Birmingham. Her testimonials are excellent, and we congratulate her on her appointment.

### Minor Appointments.

Birmingham Infirmary.—Miss Annie E. Jackson has been made Night Superintendent at this infirmary. She was trained at St. Thomas's Hospital, and has been a Sister at the Birmingham Infirmary for five years, where her services are highly appreciated.

### Everybody's Opinion.

[Correspondence on all subjects is invited, but we cannot in any way be responsible for the opinions expressed by our correspondents. No communications can be entertained if the name and address of the correspondent is not given, or unless one side of the paper only be written on.]

### NURSING IN SAN FRANCISCO.

"Policy No. 1,122" writes: I want to let English nurses know, through The Hospital, how useless it is for them to hope to get work here. There are five training schools in which girls from the age of eighteen can get training. They receive eight dollars a month for uniform during two years, and then are supposed to be perfect in every branch of nursing. One superintendent said to me, "Get away as soon as you can; San Francisco is full of nurses. I have to write all over the country to get places for the nurses trained here. I have known nurses come out and get simply stranded." I said, "I shall write your words to The Hospital"; and she answered, "Yes, do; for The Hospital goes all over the world, and it is only fair to let all nurses know. So I have to the American papers. San Francisco used to be a good field for nurses, but now the supply far exceeds the demand." It is said that nurses belonging to the agency have waited two months for a case.

### COTTAGE NURSES.

"A Reader" writes: I venture to enclose to you a cutting from a local paper in which you will read "a trained nurse is already at work in Edgmond." I see this paper regularly, and have observed that it persists in speaking of the "cottage nurses" as "trained nurses." Their experience actually consists in six months' work, half spent in the district, and half in learning midwifery. Although this district is not a rich one it is not particularly poor, but I suppose economy accounts for the employment of cottage nurses.

[We do not consider there is any economy in using partially taught persons in place of thoroughly trained ones. Of course the error made by the correspondent whose paragraph you enclose is one which others will fall into, and with perhaps better excuse for the blunder. We have always maintained that the title of nurse should not be bestowed on "cottage helps." The latter are useful women but should not be allowed to personate district nurses.—Ed. *T. H.*]

### SOUTH AFRICA AS A HEALTH RESORT.

"P. M. D." writes: Much is said and written about Africa as the "country of the future," and doctors order patients with weak lungs or throats to go to South Africa. Possibly Africa is "the country of the future," but its thinly-peopled land has been the burial ground of many pioneers. As a health resort it can scarcely be over-estimated if—and this is a very large "if"—those who come for health have a liberal supply of money, and some knowledge of the dangers to be avoided, and of the difference of climates in various parts of the colony. Cape Town is ill-drained, and in the summer (from June to September) liable to high winds called "South-easters," which brings cloud of yellow dust. The winter is very wet and cold. Hotels poor, and boarding-houses rough; therefore, accommodation such as invalids require is impossible of attainment. Ronderbosch and Wynberg are lovely suburbs, but the masses of trees make them unsuitable for lung and throat troubles. Sea Point, which is safer, has a clean and satisfactory hotel, but in April white sea fogs drive cautious invalids away. Grahamstown in April, May, and June, is good for lungs and throats, but in December, January, and February it is decidedly unhealthy, and doubtful during the other months. It is a very English town, with a good library and much sociability, so that invalids like the place. It is also fortunate in having an English doctor recently settled there. The Free State air is specially good for lung diseases, but does not suit all cases equally well, and is dangerous for those who come up direct from the coast, the high air being apt to produce hæmorrhage. The cost of board is £7 or £8 a month, milk and wine being always extras. The houses are built for summer use, and have ill-fitting doors and windows, and often no fireplaces except in the kitchen, yet the cold in South Africa is great enough for furs and thick wraps to be needful. Few people take boarders, and their accommodation is very rough; and hotels are poor and the attendance bad. The expenses of travelling are high, yet when one thinks of the distance covered this is hardly wonderful, and the trains are good, and the food on the line is fairly so. The cart and coach journeys are trying, although they may be amusing to people in good health. The sunshine and dry air of the Free States are wonderful restoratives, but Bloemfontein is dusty. In a suburb there is one English house which takes in a limited number of boarders at £10 a month, and there are two able English doctors in the town. The majority of the South African country farms are what Mrs. Crouwright Schreiner describes in her "Story of a South African Farm." Milk, butter, eggs, and vegetables are peculiarly scarce on them. During part of the year milk is 1s. a bottle (about a quart), and a cauliflower 2s. 6d., butter from 3s. to 7s. a pound. Hence living is costly. Very many invalids come to South Africa and die, despite the lovely climate, for lack of the home comforts so needful for them. Others have not sufficient means to keep them without work, and work is hard to get—in fact, a tutor or governess on a South African farm needs unusual strength allied to the appetite and digestion of an ostrich. I have seen many persons die who might have lived with proper care, which they had not the means to secure, and South Africa is a very desolate place to die in. Others, again, whose means will enable them to remain in parts suited to their special case, live and enjoy life, when at home they would be permanent invalids. The coast towns are, as a rule, delightful, but bad for throat cases. People in England do not readily grasp the great size of Africa, and doctors who have only paid it flying visits hardly seem to grasp the danger of the rapid changes. I was astonished to find pneumonia, bronchitis, diphtheria, and typhoid quite common out here until I observed that the sudden changes from the heat of day to the cold of night or the damp of sundown accounted for these illnesses. Disease makes very rapid progress here, and the dead are buried in a few hours. Yet, on the other hand, after a sharp illness recovery is more rapid than at home. Whilst the benefits of South African air cannot be over-estimated, invalids and their physicians need to guard cautiously against the dangers and difficulties that the invalid must encounter. I am one of the fortunate "recovered invalids," and should be glad to give information to others coming out, for I have tried many places and had varied experiences.

### Nurses at the Addenbrooke Hospital.

At the last quarterly meeting of the hospital Dr. MacAlister called attention to the inadequate sleeping accommodation for the probationers and nurses at the Addenbrooke Hospital, Cambridge, a fact which has been noticed in our columns in a previous issue. It appears that the same accommodation has been serving for both day and night nurses, and the undesirability of this proceeding is fully admitted by the management of the hospital. Plans for a proper scheme are now under consideration, and a special court is to be held to consider the committee's report and to decide upon some plan of action. The proposed alterations will admit of an increased number of probationers being received, thus adding to the funds of the hospital.

### Dress and Uniforms.

By a Matron and Superintendent of Nurses.

### III.

Messrs. Debenham and Freebody are showing some excellent samples of uniform which are well worth a visit of inspection. Everything supplied by so high-class a firm is in the best of taste, and their reputation is well maintained in this special branch. A linen apron with bib and straps, well

The Victoria made in every particular, can be obtained for the astonishingly low price of 1s. 6d., and this we especially recommend to probationers entering on trial who have to procure their own uniform. A very fine linen apron is correspondingly cheap at 3s. 11d. What strikes us particularly in this class of goods is the neatness and finish of the stitching, which we have no hesitation in pronouncing to be the very best we have ever seen at the price. Dainty linen sleeves are marked at 11d. per pair, and we should recommend all private nurses to procure a few of these for use at operations. Bonnets are supplied by this firm from 5s. 6d. upwards. The accompanying illustration is of a pretty shape called the "Victoria" which costs, trimmed

The Dorothy with velvet, white cap-fronts, and strings, the modest price of 8s. 6d. It can be had in all colours but black perhaps is prettiest. The "Dorcas" is a neat little shape and intended to be worn with a gossamer veil, which latter adjunct we do not recommend as it is not only useless but extravagant; the bonnet, however, can be worn equally well without.

The cloaks made by Messrs. Debenham and Freebody, it is needless to say, are admirable both in cut and style. There is one they specially recommend—the "Sister Dora." This cloak is particularly graceful, and well adapted to a nurse's requirements. They keep also a very good assortment of circular cloaks, which, in our opinion, are as suitable and pretty as any. The cap that takes our fancy most is the "Dorothy," which, as the illustration shows, is both simple and pretty; it also washes easily, as all that is required is to untie the draw string, and it becomes quite flat. A neat little bow keeps the cap in position, tied under the chin.

We would call the attention of our readers to an especially useful travelling trunk made by Fisher, 188, Strand. It is supplied in two qualities, in bright canvas with leather mountings, at 40s., or in dull canvas at 30s. It is a very useful size, and measures 30 in. by 16 in. by 13 in. A moveable slide gives a separate compartment when required for cuffs, collars, caps, and other light articles.

### Boots and Shoes.

The problem of boots and shoes is one which is always present with us in some form or another. Those members of the community who, like nurses, are constantly on their feet, will appreciate the benefit, therefore, of an article constructed on really hygienic principles. Messrs. John Hotblack, of Norwich, have favoured us with a sample of their boots and shoes, the idea of which is to preserve the big toe in its natural position. There can be no question of the comfort such sensibly-contrived articles must be to the wearer, and as many nurses suffer from tender feet, we should recommend them to give Messrs. Hotblack a trial. The accompanying illustration shows the natural position of the foot, to which the shoe is cleverly adapted.

### Materials for Dresses and Cloaks.

Messrs. Frank Murgatroyd and Co., of Bradford, are showing some delightful novelties in the way of tweeds, serges, and cloakings, which are certain to achieve a well-merited popularity. The covert coatings in shades of brown and grey would make up into the most useful and attractive costumes, and as they are of substantial consistency, will have no difficulty at this season of the year in securing purchasers. A thick black cheviot especially took our fancy, and is the very material out of which to fashion a warm and useful cloak for winter wear. The price per yard, 52 inches wide, is only 2s. 2d. There are also some very serviceable cloths, double width, at prices varying from 1s. 3d. to 4s. 9d. All the materials kept by this firm are to be relied on, and are certainly inexpensive. Orders exceeding 7s. in value are sent carriage paid to any address in the United Kingdom.

---

### ANSWERS TO CORRESPONDENTS.

Boots for District Nurses. (E.G.)—Where can I procure porpoise hide boots suitable for district nurses use?—We have ascertained from the London Shoe Company (whose advertisement in our columns please see) that they can supply boots in this material made to order at 24s. 9d. They recommend in preference, however, a waterproof boot, lined with chamois leather, which they are now making calculated to defy all weathers, and which can be had in any size, at prices ranging from 18s. 6d. upwards. The only advantage in porpoise hide boots is the fact that they do not crease. The boots recommended by the Shoe Company would be equally durable and waterproof.

### The Book World for Women and Nurses.

[We invite Correspondence, Criticism, Enquiries, and Notes on Books likely to interest Women and Nurses. Address, Editor, THE HOSPITAL (Nurses' Book World), 428, Strand, W.C.]

BLESSED ARE THE POOR. By FRANÇOIS COPPÉE. Translated from the French by WINIFRED HEATON. 1 Vol. (London: Heinemann. 1894.)

We are better acquainted with M. Coppée as a writer of verse than of prose. These two stories, however, bound together, and entitled "Blessed are the Poor" show that he is not less skilled as a prose writer than a poet. Wonderfully little is lost in the process of translation, which is admirably done, and the little book is furnished with a preface by Mr. T. P. O'Connor, which is not the least attractive portion of it. Both stories point the moral that wealth does not either bring or promote happiness. The moral is so naively apparent, and the stories are both written in so charming and simple a style as to call for no apology for the theme. The first tale is called "Restitution," and tells of a fraudulent banker who escapes from the clutches of the law, and being conscience-stricken, and anxious to ultimately justify himself in the eyes of his little son, spends many years in amassing sufficient honestly-won money to enable him to repay his creditors. When the time comes that he is in a position to refund both capital and interest, he returns to Paris and seeks out an old abbé, to whom he entrusts the payment of his debts. The point of the story is in the showing that the sudden possession of money by the creditors brings no happiness with it; whereas the loss of it had in several cases been the moral saving of several people, who were compelled to work instead of leading idle, useless lives. The companion story is entitled "The Poverty Cure," and is written on much the same lines, showing how the winning of the big prize in the International Lottery was very nearly the spiritual and intellectual undoing of a young artisan with a turn for verse writing. With the sudden accession to wealth, Albéric leaves his work and his verses, and takes to an idle life of luxury, which results in mental and physical *ennui*. Realising his danger, he inflicts upon himself a "poverty cure," which means a return to hard work and a dirty garret. In a few days Albéric is represented as free from *ennui*, and the husband of a poor but virtuous young women. Having once realised the happiness to be found in simplicity of life, he takes a small house in the country, and writes verses in praise of Zoë, his wife.

### MAGAZINES OF THE MONTH.

THE HUMANITARIAN is of fairly uniform interest in the present issue. Perhaps the most popular article will be found in Miss Florence Nightingale's "Village Sanitation in India." The substance of this paper was read before the Tropical Section of the Eighth International Congress of Hygiene and Demography held at Buda-Pesth in September. Miss Nightingale has her facts well in hand, and whilst she brings the difficulty of the subject to light the writer leaves the solution of the problem in other hands. "I earnestly trust," she says, "that those who are now bringing their learning and experience to bear upon these difficult problems will favourably view this my humble effort to throw light upon the causes of disease in India, and will use their powerful influence to improve the condition of the many millions when health, life, and happiness are involved."

CASSELL'S FAMILY MAGAZINE opens with a romantic and delightfully illustrated phantasy on "Sun Rise in the Moon." This paper, the outcome of Mr. Munro's imaginative brain, is at best of a speculative nature, but it has a certain fascination for those whose education is limited to the lighter side of astronomy. There are some clever portrait studies in Mr. J. Cuthbert Hadden's "Musicians"—we are speaking of these particularly in reference to the actual illustrations, which are both well drawn and well reproduced. The fictional matter in this month's issue is up to the usual standard, and there are several papers of much interest.

THE STRAND MAGAZINE does not appear to be sinking into that vortex towards which many of our weeklies are fast being drawn, into a receptacle for short stories, that is. Out of the fourteen articles in this month's issue, five only are of a fictional character. Two papers, the one on "Giants and Dwarfs," profusely illustrated, the other "The Dogs of Celebrities," are both interesting and entertaining.

THE SCIENTIFIC WORLD (Marcus Ward and Co., annual subscription 5s.) has lately made its first appearance amongst us, and is another proof of the hold science has on the public mind. Popular science, anyhow; for there is science and science. Our new contemporary, which lies before us for review, describes itself as a "Popular monthly review of art, science, mechanics, and manufactures." The field which it embraces is, therefore, not a limited one, and we shall follow its career with interest. It strikes one at first sight as having a leaning rather to the "manufactures" than to the sciences themselves, and is undoubtedly a medium for advertisements. But the *Scientific World* is nicely got up, printed in good type on good paper, and we wish Messrs. Marcus Ward and Co. all success in their new venture.

### Presentation.

ON Saturday, the 13th inst., the officers of the Fir Vale Workhouse assembled to present Dr. Waters, the late Resident Medical Officer, with a handsome travelling clock, as a token of their esteem and regard. The presentation was made by Dr. Hunt, who spoke as to the admirable and courteous manner in which Dr. Waters discharged his duties whilst connected with the institution. Dr. Waters also received a pretty reading lamp from the nurses.

### Where to Go.

CHRYSANTHEMUM SHOW IN THE INNER TEMPLE (entrance gate on the Embankment).—This annual exhibition opened on Thursday, 18th inst., and, by the courtesy of the Benchers, the public is admitted from 10 a.m. till dusk.

SANITARY INSTITUTE, MARGARET STREET, W.—The course of lectures commenced on October 17th with "Elementary Physics" by Mr. John Castett-Evans, F.I.C. Lectures and Demonstrations for Sanitary Officers will take place on Mondays, Wednesdays, and Fridays at 8 p.m. till December 3rd inclusive.

### Notes and Queries.

### Queries.

(16) *Training.*—If a probationer leaves a hospital at the end of her trial month, to whom should she apply for testimonials?—*F. J. S*

(17) *Hospital Nurse.*—How should I proceed to get trained as a hospital nurse in London?—*M. L. M.*

(18) *Pension.*—How can I obtain a pension from the Royal Hospital for Incurables, Putney, for a respectable woman of forty who has been a schoolmistress, but is now completely paralysed?—*E. M. B.*

(19) *Two Years.*—Can you inform me of a first-class infirmary or hospital in London where a non paying probationer can be received for a long period; the first two years to be spent entirely in ward work, not in private nursing?—*C. P. G.*

(20) *One Year.*—Where can a lady of thirty-five get one year's training in nursing?—*Nurse E. W.*

### Answers.

(16) *Training* (*F. J. S.*)—We do not understand what you mean. Of course no "testimonial" could be given for a month's service. Whatever may be the cause of the probationer's leaving, such a short period can be of no value as a "reference."

(17) *Hospital Nurse* (*M. L. M.*)—Write to the matrons of general hospitals or of workhouse infirmaries and learn their regulations, and also the date of next vacancy. You will find a list of institutions in *Burdett's Hospital Annual*, published by the Scientific Press, 428, Strand. You had better read "How to Become a Nurse," by Honnor Morten; same publishers.

(18) *Pension* (*E. M. B.*)—Write and ask the Secretary for a copy of the rules, or for information as to qualifications for pension. Doubtless there are a great many applicants at each election whose claims have to be considered. Perhaps the annual report contains the information required.

(19) *Two Years* (*C. P. G.*)—See answer to query (17) above. If a workhouse infirmary be selected you could learn from the Hon. Secretary to the Workhouse Infirmary Nursing Association in which institutions the superintendents are trained nurses, which is, of course, most essential.

(20) *One Year* (*Nurse E. W.*)—See answers to queries (17) and 19) this week.

## Figures and Tables

**Figure f1:**
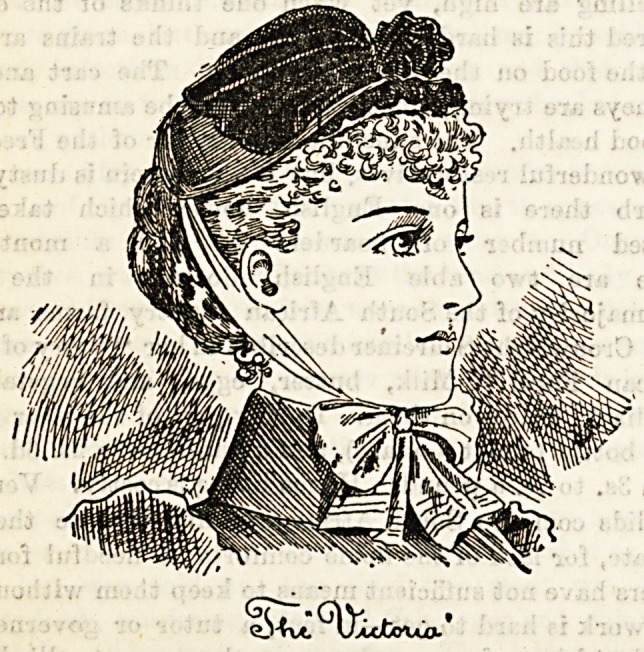


**Figure f2:**